# Congenital abnormalities in children with cancer and their relatives: results from a case-control study (IRESCC).

**DOI:** 10.1038/bjc.1993.340

**Published:** 1993-08

**Authors:** J. R. Mann, H. E. Dodd, G. J. Draper, J. A. Waterhouse, J. M. Birch, R. A. Cartwright, A. L. Hartley, P. A. McKinney, C. A. Stiller

**Affiliations:** Children's Hospital, Birmingham, UK.

## Abstract

Several studies have revealed an excess of malformations in children with certain malignancies. A few environmental causes have been identified which may damage the foetus and lead to malformation and cancer. However, most of the numerous recognised cancer/malformation syndromes are genetically determined. This report describes a case-control study of 555 newly diagnosed children with cancer and 1,110 matched controls, chosen from general practitioner lists (GP controls) and hospital admissions (H controls). Their parents were interviewed on topics of possible aetiological significance and medical records were checked to confirm reports at interview. The numbers of congenital malformations in the index and GP control children, and the relatives of the index children, the GP and H controls are described. There were more children with malformations among the cases (60/555) than among the GP controls (27/555), P < 0.001. The abnormalities in the cases included eight with specific chromosomal/genetic conditions (e.g. Down's syndrome, XY gonadal dysgenesis, Von Recklinghausen's neurofibromatosis, Goldenhar's syndrome) whereas only one GP control child had a chromosomal defect (P < 0.05). Five case children but no GP controls had neural tube defects; this is not statistically significant. No excess of malformations was found in the siblings of cases compared with GP and H control siblings. Case mothers had a small excess of malformations (22/555) compared with GP controls (8/555), P < 0.05. Among more distant relatives the results were difficult to interpret because of the relatively small numbers in the diagnostic subgroups and because of apparent under reporting in grandparents, but no striking differences were seen between case and control relatives. The excess of malformations found in children with cancer, compared with controls, without a similar excess of malformations in their close relatives may indicate that in some (perhaps very roughly one in 20) cases antenatal events may lead both to the malformation and the malignancy.


Br. J. Cancer (1993), 68, 357-363 © Macmillan Press Ltd., 1993

### Congenital abnormalities in children with cancer and their relatives: results from a case-control study (IRESCC*)

J.R. Mann[1], H.E. Dodd[1], G.J. Draper[2], J.A.H. Waterhouse[3], J.M. Birch[4], R.A. Cartwright[5], A.L. Hartley[4], P.A. McKinney[5] & C.A. Stiller[2]

[1]*The Children's Hospital, Ladywood Middleway, Birmingham B16 8ET;* [2]*Childhood Cancer Research Group, University of Oxford, Woodstock Road, Oxford OX2 6HJ;* [3]*Birmingham and West Midlands Regional Cancer Registry, Queen Elizabeth Medical Centre, Birmingham B15 2TH;* [4]*CRC Paediatric and Familial Cancer Research Group, Christie Hospital and Holt Radium Institute, Manchester M20 9BX;* [5]*Leukaemia Research Fund Centre for Clinical Epidemiology, University of Leeds, Department of Pathology, 17 Springfield Mount, Leeds LS16 9NG, UK.*

**Summary** Several studies have revealed an excess of malformations in children with certain malignancies. A few environmental causes have been identified which may damage the foetus and lead to malformation and cancer. However, most of the numerous recognised cancer/malformation syndromes are genetically determined.

This report describes a case-control study of 555 newly diagnosed children with cancer and 1,110 matched controls, chosen from general practitioner lists (GP controls) and hospital admissions (H controls). Their parents were interviewed on topics of possible aetiological significance and medical records were checked to confirm reports at interview. The numbers of congenital malformations in the index and GP control children, and the relatives of the index children, the GP and H controls are described.

There were more children with malformations among the cases (60/555) than among the GP controls (27/555), $P<0.001$. The abnormalities in the cases included eight with specific chromosomal/genetic conditions (e.g. Down's syndrome, XY gonadal dysgenesis, Von Recklinghausen's neurofibromatosis, Goldenhar's syndrome) whereas only one GP control child had a chromosomal defect ($P<0.05$). Five case children but no GP controls had neural tube defects; this is not statistically significant.

No excess of malformations was found in the siblings of cases compared with GP and H control siblings. Case mothers had a small excess of malformations (22/555) compared with GP controls (8/555), $P<0.05$. Among more distant relatives the results were difficult to interpret because of the relatively small numbers in the diagnostic subgroups and because of apparent under reporting in grandparents, but no striking differences were seen between case and control relatives.

The excess of malformations found in children with cancer, compared with controls, without a similar excess of malformations in their close relatives may indicate that in some (perhaps very roughly one in 20) cases antenatal events may lead both to the malformation and the malignancy.

In Western developed countries childhood cancers represent only about 0.5% of all human cancers and differ in many ways from those in adults. In these countries about one third are acute leukaemias, 10-15% lymphomas, 28% central nervous system tumours and 15% various types of embryonic tumours. On the other hand, adult cancers are mostly carcinomas, 'associated with prolonged exposure to a hostile environment' (Dodet & Lenoir, 1990). About 40% of paediatric malignancies in Western countries develop before the age of 5 years and the few environmental causes so far identified have mostly exerted their effect ante-natally and may also lead to malformation. Examples have been antenatal exposure to diethylstilboestrol causing adenocarcinoma of the vagina, dysplasia of vagina and cervix and maldescent and tumour of the testis (Herbst *et al.*, 1971; Bibbo *et al.*, 1977; Brown *et al.*, 1986); antenatal diagnostic irradiation and subsequent cancers especially leukaemias (Stewart *et al.*, 1956) or mental retardation (Mole, 1979); antenatal exposure to phenytoin causing the foetal hydantoin syndrome and neuroblastoma (Allen *et al.*, 1980; Ehrenbard & Chaganti, 1981); and adrenal carcinoma (Hornstein *et al.*, 1977) or hepatoblastoma (Khan *et al.*, 1979) associated with foetal alcohol syndrome caused by antenatal exposure to alcohol (though the associations between childhood cancer and antenatal exposure to either alcohol or phenytoin are based only on case reports, not controlled studies).

On the whole genetically determined factors seem to be more important than environmental agents in the aetiology of childhood cancers, although they may interact with each other. Knudson proposed a 'two mutation' or 'two hit' hypothesis for the development of retinoblastoma (Knudson, 1971; Hethcote & Knudson, 1978; Knudson, 1978) which has since been shown by DNA technology to be correct (Cavenee *et al.*, 1983) and may apply to several other childhood cancers too. However, although a large number of specific anomalies has been described in association with certain childhood cancers (Schimpke, 1978; Knudson, 1986; Dodet & Lenoir, 1990) such as sporadic aniridia, genito-urinary anomalies and mental retardation with Wilms' tumour due to chromosome 11p 13 deletions (Riccardi *et al.*, 1978) there have also been reports of an excess of various less specific anomalies in children with Wilms' tumour (Miller *et al.*, 1964), germ cell tumours (Birch, 1980; Birch *et al.*, 1982; Johnston *et al.*, 1986), rhabdomyosarcomas (Ruymann *et al.*, 1988) and liver tumours (Mann *et al.*, 1990).

The purposes of the investigation described in this report were to quantify the association of congenital malformations with childhood cancers, and, by studying the incidence of malformations in relatives, to determine to what extent these were inherited and to what extent they might represent new mutations. In addition, family pedigrees were examined for evidence of known and hitherto unrecognised cancer-associated syndromes.

### Methods

The parents of 555 children who were newly diagnosed to have cancer and resident in the West Midlands, North West and Yorkshire Health Authority Regions in England were interviewed with a standard questionnaire. Each case child was matched for age and sex with two control children selected from general practitioner lists, designated GP controls (GPC), and hospital admissions, designated hospital

Correspondence: J.R. Mann.

*IRESCC Group: The authors and C.C. Bailey, A.H. Cameron, R.H.A. Campbell, S.C. Cartwright, J.J. Corkery, D. Deakin, P. Gornall, P.A. Hopton, H.B. Marsden, P.H. Morris Jones, D. Pearson, R. Swindell and J. Williams.

Received 13 July 1992; and in revised form 22 February 1993.

controls (HC). The detailed methodology, which has been described elsewhere (Birch et al., 1985), included the verification of information gained at interview by reference to medical records, both of cases and controls. This process included examination of the obstetric records and the general practitioners' records for *all* cases and controls, irrespective of whether a malformation in the index child had been reported at interview. Appropriate research ethical committee approvals were obtained.

Congenital malformations in the index children and their relatives were defined as those defects listed in Chapter XIV of The Ninth Revision of the International Classification of Diseases (WHO, 1977) and also neurofibromatosis, congenital deafness and congenital and infantile hernia. For the index children we included, in addition, sacral dimples and tufts of hair over the lumbo-sacral spine. In studying the incidence of congenital malformations in the index children, only the cases and GPC were compared, since children with major defects were not considered eligible to be HC (Birch et al., 1985). For siblings, parents, grandparents and 'other relatives' (uncles, aunts, half uncles, half aunts and cousins), comparisons were made between the numbers of malformations in these relatives of case, GPC and HC children. For index children, siblings and parents, only malformations which had been confirmed by inspection of medical records were used in the analysis but for grandparents and 'other relatives', whose records were often unobtainable, malformations reported at interview were analysed. For all relatives we excluded birthmarks, naevi, skin tags, café au lait spots and any condition of dubious significance. Anomalies in stillborn relatives were included in the analyses. Maternal half siblings were included in the sibling totals.

Tables for case-control comparisons were produced using SPSS-X (1985). Yates correction was included in the analysis. McNemar's matched pair analysis was used for comparison of congenital anomalies in the index cases and GP control children and also for comparison of case and control parents (Fleiss, 1973).

### Results

The total numbers of individuals with congenital malformations among the case and control groups are shown in respect of the index children, their siblings, parents, grandparents and other relatives in Table I.

### *Index children*

The numbers of children with congenital malformations in the cases and GP controls are shown for each diagnostic group in Table II. There were more case than control children with malformations in nine of the 14 diagnostic groups: however the only group to show a significant difference was the germ cell tumours, as previously reported (Johnston et al., 1986). For all groups together a total of 60 case children had malformations, compared with 27 in the control group, a highly significant difference, $P<0.001$. The excess of malformations in all the patients with embryonal tumours grouped together (Wilms', neuroblastoma, retinoblastoma, hepatoblastoma and germ cell tumours) was statistically significant (C18, GP controls 7, $P<0.05$).

In Table III the nature of the malformations recorded in the case children is described for each diagnostic group and in Table IV the malformations recorded in the GP controls are listed. The anomalies observed were of many types and affected most organs and systems.

The commoner anomalies are summarised as a case-control comparison in Table V. A total of eight case children had chromosomal or genetic conditions of which a number are already known to pre-dispose to malignancy. Of these, Down's syndrome was present in three children with leukaemia, and a child with X-linked XY gonadal dysgenesis had gonadoblastoma (previously reported, Mann et al., 1983). Also, a boy with skin lesions and IgA deficiency had adenocarcinoma of the caecum and later died of non-Hodgkin's lymphoma; his brother, who also had skin lesions, IgA deficiency and cystic hygroma had glioblastoma multi-

**Table I** Numbers of relatives with congenital malformations

| | *Case* | *GP control* | *Hospital control* |
|---|---|---|---|
| Number of index children with malformations[a] | 555 | 555 | 555 |
| | 60 (10.8%) | 27 (4.9%) | – |
| Number of siblings studied with malformations[a] | 843 | 779 | 814 |
| | 35 (4.2%) | 38 (4.9%) | 42 (5.2%) |
| Number of parents studied with malformations[a] | 1,086 | 1,090 | 1,078 |
| | 28 (2.7%) | 15 (1.4%) | 18 (1.7%) |
| Number of grandparents studied with malformations[b] | 2,113 | 2,108 | 2,149 |
| | 17 (0.8%) | 28 (1.3%) | 14 (0.7%) |
| Number of other relatives studied with malformations[b] | 7,836 | 8,199 | 7,522 |
| | 205 (2.6%) | 175 (2.1%) | 205 (2.7%) |

[a]Only malformations confirmed by inspection of medical records were included. [b]All malformations reported at interview were included.

**Table II** Number of index children with congenital malformations

| *Diagnostic group* | *No.* | *No. of case children with malformations* | *No. of GP control children with malformations* | *Significance* |
|---|---|---|---|---|
| Acute lymphoblastic leukaemia | 148 | 11 | 8 | NS |
| Other leukaemias | 23 | 3 | 0 | NS |
| Hodgkin's disease | 32 | 4 | 1 | NS |
| Other lymphomas | 31 | 3 | 4 | NS |
| Central nervous system tumours | 78 | 10 | 3[a] | NS |
| Soft tissue sarcoma | 43 | 6 | 1 | NS |
| Bone tumours | 30 | 3 | 2 | NS |
| Wilms' tumour | 32 | 5 | 1 | NS |
| Neuroblastoma | 35 | 4 | 4 | NS |
| Retinoblastoma | 6 | 0 | 1 | NS |
| Hepatoblastoma | 6 | 2 | 0 | NS |
| Germ cell tumours | 41 | 7 | 1 | $P<0.05$ |
| Epithelial tumours | 22 | 1 | 0 | NS |
| Other neoplasms | 28 | 1 | 1 | NS |
| Total | 555 | 60 | 27 | $P<0.001$ |

NS = $P>0.05$ – not significant. [a]Four were reported in the paper by Birch et al. (1990) but one was a hairy mole/naevus and therefore was excluded from this paper.

**Table III** Congenital malformations in case children

| *Diagnosis* | *Description of malformation* |
|---|---|
| Acute lymphoblastic leukaemia | Down's syndrome (Trisomy 21)<br>Down's syndrome mosaic (80% of karyotype 47XY)<br>Goldenhar's syndrome<br>Coarctation of aorta and bilateral valgus deformity of feet<br>Undescended testes<br>Glandular hypospadias[a]<br>Encysted hydrocoele of spermatic cord<br>Hip deformity<br>Large skull[a]<br>Ptosis left eyelid<br>Bowed legs - internal tibial torsion, inguinal hernia[a] |
| Other leukaemias | Down's syndrome<br>Spina bifida and hydrocephalus<br>Varus deformity of foot |
| Hodgkin's disease | Ventricular septal defect<br>Pyloric stenosis<br>Talipes and malformed toe<br>Supernumerary thumb |
| Other lymphomas | Hypertrophy and port wine stain of arm, and naevi of trunk<br>Undescended testis<br>Polycystic kidneys |
| Central nervous system | Bilateral undescended testes<br>Undescended testis<br>Unstable hips<br>Dislocated hip<br>Talipes equino varus<br>Bilateral metatarsus varus<br>Scoliosis, squint,[a] pigeon chest<br>Epicanthic folds[a]<br>Sternomastoid tumour[a]<br>Left auricular sinus |
| Soft tissue sarcoma | Tongue tie<br>Deep sacral dimple and raised AFP in pregnancy<br>Hypospadias and club foot<br>Undescended testis<br>Absent phalanx left 5th finger and bilateral clinodactyly<br>Over-riding 2nd and 3rd toes left foot and right 4th toe sticks forward[a] |
| Bone tumours | Meningomyelocoele<br>Absent left kidney and ureter[a]<br>Low set ears, 'clicking' hips, haemangioma of left leg, genitals and buttocks |
| Wilms' tumour | Plagiocephaly<br>Dislocated hip<br>Bilateral talipes equino varus, low set ears, short palpebral fissures[a]<br>Kyphoscoliosis, short neck, multiple spinal anomalies, absent ribs, large tongue, small fontanelle, spina bifida occulta, accessory nipple and hirsutism<br>Sinus and spina bifida at S1 |
| Neuroblastoma | Cleft palate<br>Patent ductus arteriosus<br>Tuft of hair over lumbar spine<br>Sacral dimple[a] |
| Hepatoblastoma | Ventricular septal defect<br>Small penis and red pigmentation of arm |
| Germ cell tumours | Heart murmur? VSD<br>Cervical meningocoele<br>Bilateral undescended testes<br>X-linked XY gonadal dysgenesis<br>Duplex kidney with reflux and pyelonephritis[a]<br>Right metatarsus varus[a]<br>Bilateral bat ears[a] |
| Epithelial tumours (carcinoma of caecum and later non-Hodgkin's lymphoma) | Skin lesions, IgA deficiency, (brother with skin lesions, IgA deficiency, cystic hygroma, glioma and carcinoma of rectum) |
| Multiple large plexiform neurofibromas | Von Recklinghausen's neurofibromatosis |

[a]Condition which was noted from inspection of medical records, not reported at interview.

forme and carcinoma of the rectum. These boys have been reported elsewhere (Al Sheyyab *et al.*, 1993). A child with von Recklinghausen's neurofibromatosis had multiple large plexiform neurofibromas, a child with polycystic kidneys had a lymphoma and a child with Goldenhar's syndrome had leukaemia. In contrast, only one child in the GP control group had a chromosomal/genetic disorder, which was a ring chromosome 16/17 associated with low set ears and talipes. The case/control excess for chromosomal/genetic disorders was 8 to 1. For neural tube defects (including spina bifida occulta) the excess was 5 to 0; this difference is not statistically significant ($P > 0.05$). The neural tube defects were

**Table IV** Congenital malformations in GP control children

| *Description of malformations* | *No. of GP control children affected* |
|---|---|
| Pigeon chest[a] | 1 |
| Hypospadias | 1 |
| Peno-scrotal webbing[a] | 1 |
| Umbilical hernia and pylorospasm | 1 |
| Metatarsus varus (± other abnormalities of legs/feet) | 3 |
| Metatarsus varus and bilateral ptosis | 1 |
| Valgus deformity of feet (and knees in 1) | 3 |
| Talipes | 1 |
| Dislocated hip(s) (and umbilical hernia in 1) | 3 |
| Sternomastoid tumour | 1 |
| Undescended testis (testes)[a] | 2 |
| Poorly developed skull bones, occipital narrowing, haemangioma buttock | 1 |
| Bilateral trigger thumb[a] | 1 |
| Bilateral genu valgus | 1 |
| Webbing 1st and 2nd toes both feet | 1 |
| Tongue tie[a] | 2 |
| Ring chromosome 16/17, low set ears, talipes | 1 |
| Sacral dimple | 1 |
| Bilateral pre-auricular sinuses | 1 |

[a]Condition which was noted from inspection of medical records, not reported at interview

**Table V** Case-control comparison of malformations in index children

| *Type of malformation* | *No. of cases affected* | *No. of GP controls affected* |
|---|---|---|
| Chromosomal anomalies | 3 | 1 |
| Single-gene disorders | 5 | 0 |
| Neural tube defects (including spina bifida occulta) | 5 | 0 |
| Sacral dimple/hair | 3 | 1 |
| Congenital heart disease/murmur | 5 | 0 |
| Hypospadias | 2 | 1 |
| Undescended testicle(s) | 6 | 2 |
| Positional hip, leg and foot deformities | 15 | 13 |

present in cases with leukaemia (1), Ewing's tumour (1), Wilms' tumour (2) and germ cell tumour (1). In one of these the spina bifida occulta was part of a syndrome of multiple abnormalities (Wilms' tumour – see Table III). When chromosomal/genetic disorders were excluded there remained a statistically significant case/control excess of abnormalities (52 in cases and 26 in controls, $<0.01$). Congenital heart disease/murmur was present in five cases but no controls; undescended testicle was commoner (though not significantly) in cases than controls. No significant case/control difference was present for positional hip, leg and foot deformities.

*Siblings and parents*

There were no differences between case and control groups in the proportions of siblings with malformations either for the groups as a whole or for the diagnostic sub-groups. Chromosomal/genetic anomalies were present in two case siblings (Down's syndrome, Prader-Willi syndrome), two HC siblings (both Down's syndrome) and three GPC siblings (two Turner's syndrome, one Down's syndrome). Neural tube defects were present in three case, three GPC and eight HC siblings. In two case families more than one sibling had congenital anomalies compared with 0 GPC and two HC families.

There was a significant difference for the numbers of mothers with anomalies in the case group as a whole when compared with the GPC group (22/555 *vs* 8/555, $P<0.05$). However, the difference between case and HC mothers (22/555 *vs* 14/555) did not reach statistical significance. Seven case fathers, seven GPC fathers and four HC fathers were affected. The numbers of neural tube defects found in case and control parents were similar (two case, three GPC, one HC). A father of a child with a germ cell tumour had bilateral cystic kidneys and hypergammaglobulinaemia (Figure 1) and the mother of a case child with neurofibromatosis had a hare lip, numerous birthmarks and naevi and an epithelial polyp; one HC parent also had neurofibromatosis.

*Grandparents and other relatives*

There were no significant differences between cases and controls in the total numbers of malformations reported at interview in these relatives. However, there appears to have been some under-reporting of malformations in grandparents, as the proportions with reported malformations among these relatives were substantially lower than among the other groups.

Among the other relatives group (uncles, aunts, cousins, half-aunts and uncles) there were similar percentages of individuals with malformations in the case and control groups overall. However, a statistically significant excess of congenital anomalies was found in case compared with GPC relatives in the acute lymphoblastic leukaemia diagnostic subgroup (49/1943 *vs* 33/2413, $P<0.01$) and specifically an excess of neural tube defects was reported in the case relatives (cases 8/1943, GP controls 1/2413, $P<0.05$). The soft tissue sarcoma group showed an excess of anomalies between case and GP control relatives (24/256 *vs* 10/585, $P<0.01$) as did the neuroblastoma group (18/416 *vs* 13/648, $P<0.05$). Significantly more anomalies in case than HC relatives were found in the Wilms' tumour group (16/449 *vs* 4/618, $P<0.01$), and in the bone tumour group (15/565 *vs* 4/492, $P<0.05$), but significant excesses were also found in HC *vs* case relatives in the Hodgkin's disease group (18/356 *vs* 10/617, $P<0.05$) and in GPC *vs* case relatives in the central nervous system tumours group (46/959 *vs* 30/1104, $P<0.05$). These results are difficult to interpret because of the large number of statistical tests that have been carried out. We report these findings for the sake of completeness, but would not attach any weight to them unless similar findings are reported from independent studies. In analysing these rates we have used chi-square tests ignoring both the

**Figure 1** Pedigree of a boy with a teratoma.

matching of cases and controls and the possible correlations between relatives. This is a further reason for treating these results with some caution.

*Family pedigrees*

Few pedigrees showed familial patterns of malformation and cancer. Those which were of interest included X-linked XY gonadal dysgenesis (Mann *et al.*, 1983) and brothers with colorectal carcinoma and also, respectively, non-Hodgkin's lymphoma and glioma (Al Sheyyab *et al.*, 1993). Also, there were several individuals with neural tube defects in the family of a boy who had Goldenhar's syndrome and acute lymphoblastic leukaemia (Figure 2) and polycystic kidneys, neurofibromatosis, cerebellar tumour, diabetes and fronto-nasal dysplasia in the relatives of a boy with teratoma (Figure 1).

### Discussion

There have been a number of reports that children with cancer have a higher incidence of malformations than do healthy children and many specific malformation/cancer syndromes and conditions predisposing to malignancy have been described (Schimpke, 1978; Knudson, 1986; Dodet & Lenoir, 1990). The genetic basis for many of these is now understood (Yunis, 1983; Green, 1988).

Miller *et al.* (1964) described congenital malformations in 66 (15%) of 440 cases of Wilms' tumour, including aniridia, microcephaly, mental retardation, hemihypertrophy, naevi, urinary-tract anomalies, gonadal anomalies and miscellaneous other defects. Thirteen per cent of 369 children with germ cell tumours reported by Fraumeni *et al.* (1973) had congenital malformations and Birch *et al.* (1982) drew particular attention to an excess of neural tube defects in children with germ cell tumours and their siblings. Among 115 children who died of rhabdomyosarcoma 37 (32%) were found at autopsy to have malformations of the central nervous, genitourinary, gastrointestinal and cardiovascular systems (Ruymann *et al.*, 1988); among them were single cases of Rubinstein-Taybi syndrome, neurofibromatosis, horseshoe kidney, hemihypertrophy and Arnold-Chiari malformation. Also of 42 children with liver tumours nine (21%) had congenital defects or other possibly related features, including hemi-hypertrophy, Beckwith-Wiedemann syndrome, renal dysplasia, undescended testis, tyrosinosis, neonatal hepatitis, bifid ureters and a family history of polyposis coli (Mann *et al.*, 1990).

Most such reports of an excess of malformations among children with cancer have used figures from normal populations for comparison. However, there are many difficulties inherent in using population figures as was described by Leck (1983). For example improved neonatal care may lead to an increase in the prevalence of malformations because more malformed infants may survive, while the overall incidence would be unaffected. Incomplete ascertainment is a problem, particularly for minor malformations and for stillbirths and neonatal deaths. Diagnoses may be inaccurate or malformations diagnosed may not be notified. Active programmes for ante-natal diagnosis and abortion of malformed foetuses can reduce the incidence of certain malformations, notably neural tube defects and Down's syndrome. Malformation rates are affected by social and other factors such as race, maternal age and parity, socio-economic status and parental consanguinity. The proportion of British children born with 'lethal and handicapping' malformations was about 2.5% using data based on work in Birmingham in the 1950's (Leck *et al.*, 1968) and other British sources (Leck, 1983), but less serious malfomations were not included in this figure. Data from the National Children's Bureau 1958 Cohort Study and other sources suggested that in the 1960's between 3 and 4% of live and stillborn infants had serious defects (Wells, 1978).

It is thus clear than an assessment of the relevance of any apparent excess number of malformations in children with cancer and their relatives is best made by a case-control study with contemporary well-matched controls. Therefore, for our study we used two types of control for each case: a

**Figure 2** Pedigree of a boy with Goldenhar's syndrome and acute lymphoblastic leukaemia.

child registered with the same general practice as the case (GP control) and a child admitted to hospital for an acute medical or surgical condition other than cancer (hospital control), both controls matched for age and sex with the case. As we specifically excluded as hospital controls children with conditions known to be associated with malignancy and certain other major malformations and diseases, the case-control comparisons for the index children could be made only between the cases and the GP control children. However, we did not consider it necessary to exclude the relatives of hospital controls from the case-control analyses for relatives.

The proportions of children with malformations among the GP controls (4.9%) and the siblings of the cases (4.2%), GP controls (4.9%) and hospital controls (5.2%) were similar. The malformations included the 'lethal and handicapping' conditions which affected 2.5% of the populations reviewed by Leck (1983) and also many less serious conditions as listed in Chapter XIV of ICD9 (WHO, 1977). Medical records were examined specifically for malformations and only those recorded in them were included in the analyses. Therefore the excess of malformations in the case children appears to be a genuine finding, particularly in view of the considerable excess of chromosomal/genetic anomalies, neural tube defects and other serious conditions. If the 'natural' incidence of malformations in the childhood population in the three regions was about 5%, then the excess malformations which can be presumed to be associated with the malignancies was just over 5% i.e. about one in twenty children with cancer may have a cancer/malformation syndrome, the cancer and the malformation perhaps sharing the same aetiology at molecular genetic level. This has already been shown to be the case for certain cancer/malformation syndromes, for example Wilms' tumour/aniridia (Riccardi *et al.*, 1978). Study of the family pedigrees of our cases showed that very few of the children with cancer and malformations had similarly affected relatives. Thus in the majority the event leading to both the cancer and the malformation can be presumed to have occurred in the ovum or sperm or after fertilisation. Ante-natal irradiation did not appear to be responsible, and all the mothers' pelvic X-rays were performed late in pregnancy.

The excess of malformations in the case children was not confined to certain diagnostic sub-groups but was evident in nearly all of them, although did not reach statistical significance except in the germ cell tumours sub-group.

The case-control comparisons for the numbers of malformations in parents, grandparents and other relatives showed no striking differences, with the possible exception that significantly more mothers of cases had malformations than did mothers of GP controls; this result may have occurred by chance since the differences between mothers of cases and Hospital Controls and the comparisons for diagnostic sub-groups were not significant. Also the numbers of affected individuals were small. It is interesting but perhaps not surprising that the percentages of parents, grandparents and other relatives with malformations were substantially lower than the figures for the index children and their siblings. Two explanations could account for this. Firstly, individuals with 'lethal and severely handicapping' malformations would have been unlikely to have achieved parenthood, grandparenthood etc. Leck *et al.* (1983) reported that such malformations affected 2.5% of persons in British populations. This figure added to the 34/2176 = 1.6% of control (HP + GPC) parents with malformations gives 4.1%, quite similar to the rates among GP control index children and siblings of cases and controls. Likewise, in the 'other relatives' group, the figure of 2.5% added to the 380/15721 = 2.4% of control (HC + GPC) 'other relatives' gives 4.9%. Secondly, the mothers of the index children, from whom the data were obtained at interview, were likely to have been less well informed about malformations (especially minor ones) in their more distant relatives than in their children, i.e. there was probably an element of under-reporting, mainly for grandparents.

Neural tube defects had occurred in paternal and maternal relatives of the child with Goldenhar's syndrome (oculo-auriculo-vertebral dysplasia, coloboma of the eyelid, dermoid of conjunctiva, accessory auricular appendages, hypoplastic zygomatic arches) and acute lymphoblastic leukaemia (Figure 2). The relationship between these conditions is unclear and so is that between the various conditions in the pedigree of a child with a teratoma (Figure 1).

Our study suggests that the excess of malformations found in children with cancer, compared with controls, without a similar excess of malformations in their close relatives may indicate that in some cases, perhaps about one in twenty, antenatal events may lead both to the malformation and the malignancy.

We thank the parents of the children included in the study and the many general practitioners, consultants and nurses in the three Regions who assisted us. Also the Cancer Research Campaign, the Leukaemia Research Fund, the DHSS, The Scottish Home and Health Department, the Special Trustees for the former United Birmingham Hospitals Trust Funds and the Special Trustees of Leeds Western Health Authority for financial support. We thank Dr H.G. Frank, Dr E. Hill and many other paediatricians, surgeons and radiotherapists whose patients were included, the medical records officers and Cancer Registries in the three regions for their help. We thank Dr D.I.K. Evans, Dr F.G.H. Hill and Professor M.F. Greaves for haematological confirmation of diagnoses, OPCS for access to death certificates, Mrs P. Brown, Mr R.W. Boyko, Mrs C. Christmas, Mrs P. Dilworth, Miss C. Kite, Miss F.M. Landells, Mrs A. Mainwaring, Miss G. Mason, Mrs J. Olden, Mrs E.M. Roberts, Dr M. Potok, Mrs S. Warner and Mr D. Winterborn for secretarial, statistical and computing assistance, Rank Xerox for photocopying, Systime Ltd for the gift of a computer, the University of Leeds for the use of the Amdahl computer and Mrs K. Evans for typing the manuscript.

### References

AL-SHEYYAB, M., MUIR, K.R., CAMERON, A.H., RAAFAT, F., PINCOTT, J.R., PARKES, S.E. & MANN, J.R. (1993). Malignant epithelial tumours: a population-based study. *Med. Ped. Onc.* (in press).

ALLEN, R.W., BUCHLER, B., OGDEN, B., BENTLEY, P.G. & JUNG, A.L. (1980). Fetal hydantoin syndrome, neuroblastoma and haemorrhagic disease. *Pediatr. Res.*, **14**, 530.

BIBBO, M., GILL, W.B., AZIZI, F., BLOUGH, R., FANG, V.S., ROSENFIELD, R.L., SCHUMACHER, G.F.B., SLEEPER, K., SONEK, M.G. & WIED, G.L. (1977). Follow-up study of male and female offspring of DES-exposed mothers. *Obstet. Gynecol.*, **49**, 1–8.

BIRCH, J.M. (1980). Anencephaly in stillborn sibs of children with germ cell tumours. *Lancet*, **1**, 1257.

BIRCH, J.M., MARSDEN, H.B. & SWINDELL, R. (1982). Pre-natal factors in the origin of germ cell tumours of childhood. *Carcinogenesis*, **3**, 75–80.

BIRCH, J.M., MANN, J.R., CARTWRIGHT, R.A., DRAPER, G.J., WATERHOUSE, J.A.H., HARTLEY, A.L., JOHNSTON, H.E., MCKINNEY, P.A., STILLER, C.A. & HOPTON, P.A. (1985). The inter-regional epidemiological study of childhood cancer (IRESCC). Study design, control selection and data collection. *Br. J. Cancer*, **52**, 915–922.

BIRCH, J.M., HARTLEY, A.L., TEARE, M.D., BLAIR, V., MCKINNEY, P.A., MANN, J.R., STILLER, C.A., DRAPER, G.J., JOHNSTON, H.E., CARTWRIGHT, R.A., & WATERHOUSE, J.A.H. (1990). The inter-regional epidemiological study of childhood cancer (IRESCC): case-control study of children with central nervous system tumours. *Br. J. Neurosurg.*, **4**, 17–26.

BROWN, L.M., POTTERN, L.M. & HOOVER, R.M. (1986). Prenatal and perinatal risk factors for testicular cancer. *Cancer Res.*, **46**, 4812–4816.

CAVENEE, W.K., DRYJA, T.P., PHILLIPS, R.A., BENEDICT, W.F., GODBOUT, R., GALLIE, B.L., MURPHREE, A.L., STRONG, L.C. & WHITE, R.C. (1983). Expression of recessive alleles by chromosomal mechanisms in retinoblastoma. *Nature*, **305**, 779–784.

DODET, B. & LENOIR, G.M. (1990). Aetiology of childhood cancers. *Ann. Nestle*, **48**, 117–124.

EHRENBARD, L.T. & CHAGANTI, R.S.K. (1981). Cancer in the fetal hydantoin syndrome. *Lancet*, **2**, 97.

FLEISS, J.S. (1973). *Statistical Methods for Rates and Proportions*. John Wiley & Sons, New York.

FRAUMENI, J.F., LI F.P. & DALAGER, N. (1973). Teratomas in children: epidemiologic features. *J. Natl Cancer Inst.*, **51**, 1425–1430.

GREEN, A.R. (1988). Recessive mechanisms of malignancy. *Br. J. Cancer*, **58**, 115–121.

HERBST, A.L., COLE, P., CARLTON, T., ROBBAY, S.T. & SCULLY, R.E. (1971). Adenocarcinoma of the vagina: association of maternal stilbestrol therapy with tumor appearance in young women. *New Engl. J. Med.*, **284**, 878–881.

HETHCOTE, H.W. & KNUDSON, A.G. (1978). Model for the incidence of embryonal cancers: application to retinoblastoma. *Proc. Natl Acad. Sci. USA*, **75**, 2453–2457.

HORNSTEIN, L., CROWE, C. & GRUPPO, R. (1977). Adrenal carcinoma in a child with history of fetal alcohol syndrome. *Lancet*, **2**, 1292–1293.

JOHNSTON, H.E., MANN, J.R., WILLIAMS, J., WATERHOUSE, J.A.H., BIRCH, J.M., CARTWRIGHT, R.A., DRAPER, G.J., HARTLEY, A.L., MCKINNEY, P.A., HOPTON, P.A. & STILLER, C.A. (1986). The Inter-Regional Epidemiological Study of Childhood Cancer (IRESCC): a case-control study in children with germ cell tumours. *Carcinogenesis*, **7**, 717–722.

KHAN, A., BADER, J.L., HOY, G.P. & SINKS, L.F. (1979). Hepatoblastoma in a child with fetal alcohol syndrome. *Lancet*, **1**, 1403–1404.

KNUDSON, A.G. (1971). Mutation and cancer: statistical study of retinoblastoma. *Proc. Natl Acad. Sci. USA*, **68**, 820–823.

KNUDSON, A.G. (1978). Retinoblastoma: a prototype hereditary neoplasm. *Semin. Oncol.*, **5**, 57–60.

KNUDSON, A.G. (1986). Genetics of human cancer. *Ann. Rev. Genet.*, **20**, 231–251.

LECK, I., RECORD, R.G., MCKEOWN, T. & EDWARDS, J.H. (1968). The incidence of malformations in Birmingham, England, 1950–59. *Teratology*, **1**, 263–280.

LECK, I. (1983). Fetal malformations. In *Obstetrical Epidemiology* Barron, S.L. & Thomson, A.M. (eds). Academic Press: London, New York, pages 263–318.

MANN, J.R., CORKERY, J.J., FISHER, H.J.W., CAMERON, A.H., MAYEROVA, A., WOLF, U., KENNAUGH, A.A. & WOOLEY, V. (1983). The X-linked recessive forms of XY gonadal dysgenesis with a high incidence of gonadal germ cell tumours: clinical and genetic studies. *J. Med. Genet.*, **20**, 264–270.

MANN, J.R., KASTHURI, N., RAAFAT, F., PINCOTT, J.R., PARKES, S.E., MUIR, K.R., INGRAM, L.C. & CAMERON, A.H. (1990). Malignant hepatic tumours in children: incidence, clinical features and aetiology. *Paed. & Perin. Epidem.*, **4**, 276–289.

MILLER, R.W., FRAUMENI, J.F. & MANNING, M.D. (1964). Association of Wilms' tumour with aniridia, hemihypertrophy, and other congenital malformations. *N. Eng. J. Med.*, **270**, 922–927.

MOLE, R.H. (1979). Radiation effects on prenatal development and their radiological significance. *Br. J. Radiol.*, **52**, 89–101.

RICCARDI, V.M., SUJANSKY, E., SMITH, A.C. & FRANCKE, U. (1978). Chromosomal imbalance in the aniridia-Wilms' tumour association: 11p interstitial deletion. *Pediatr.*, **61**, 604–610.

RUYMANN, F.B., MADDUX, H.R., RAGAB, A., SOULE, E.H., PALMER, N., BELTANGADY, M., GEHAN, E.A. & NEWTON, W.A. (1988). Congenital anomalies associated with rhabdomyosarcoma: an autopsy study of 115 cases. A report from the Intergroup Rhabdomyosarcoma Study Committee. *Med. Pediatr. Oncol.*, **16**, 33–39.

SCHIMPKE, R.N. (1978). *Genetics and Cancer in Man*, Churchill Livingstone, Edinburgh, London and New York.

STEWART, A., WEBB, A., GILES, D. & HEWITT, D. (1956). Malignant disease in childhood and diagnostic irradiation in utero. *Lancet*, **2**, 447.

WELLS, N. (1978). Studies of Current Health Problems No. 63: Birth impairments. Office of Health Economics, White Crescent Press Ltd, Luton, page 6.

WHO (1977). ICD9 Manual of the International Statistical Classification of Diseases, Injuries and Causes of Death. Ninth Revision. World Health Organisation, Geneva.

YUNIS, J.J. (1983). The chromosomal basis of human neoplasia. *Science*, **221**, 227–236.


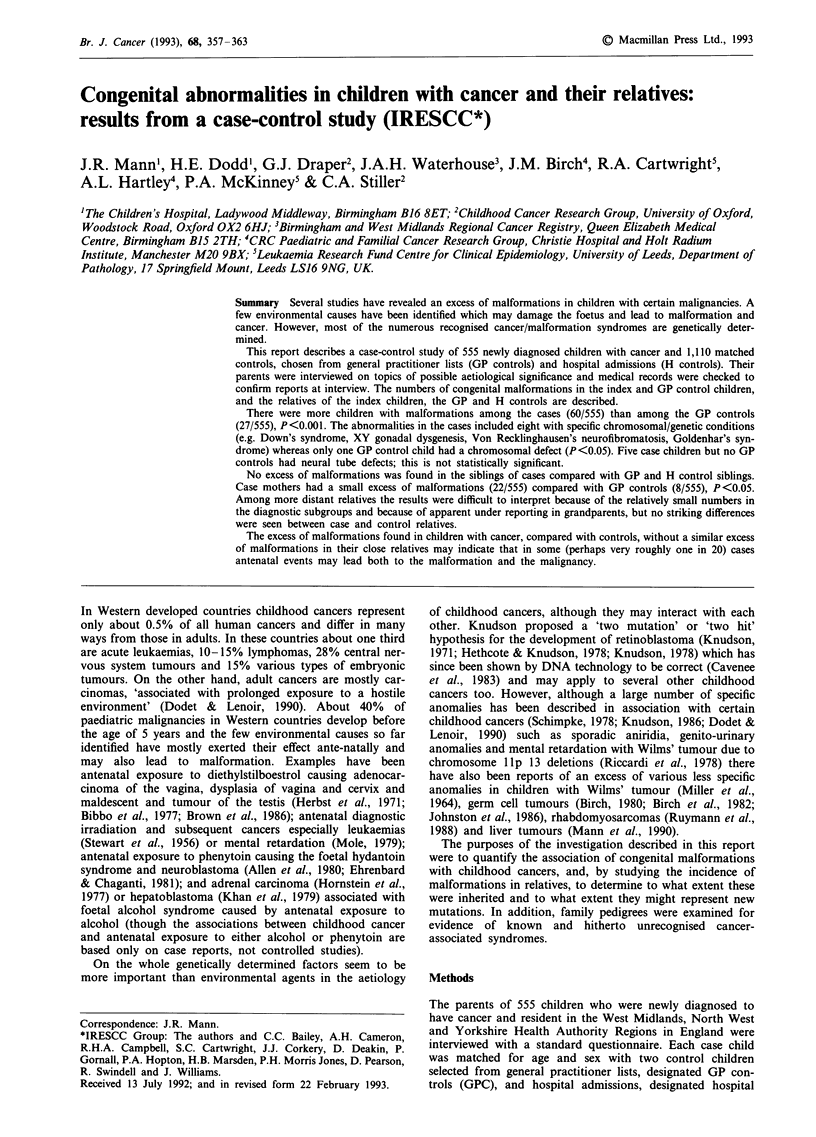

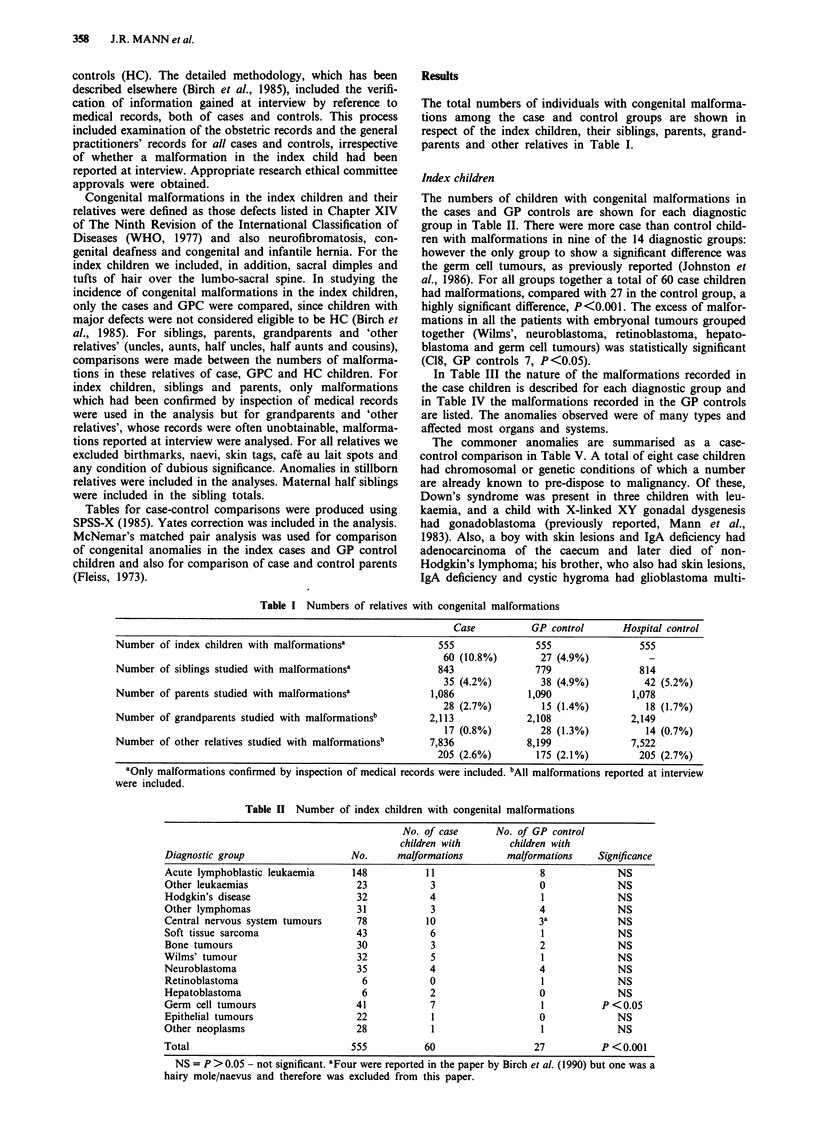

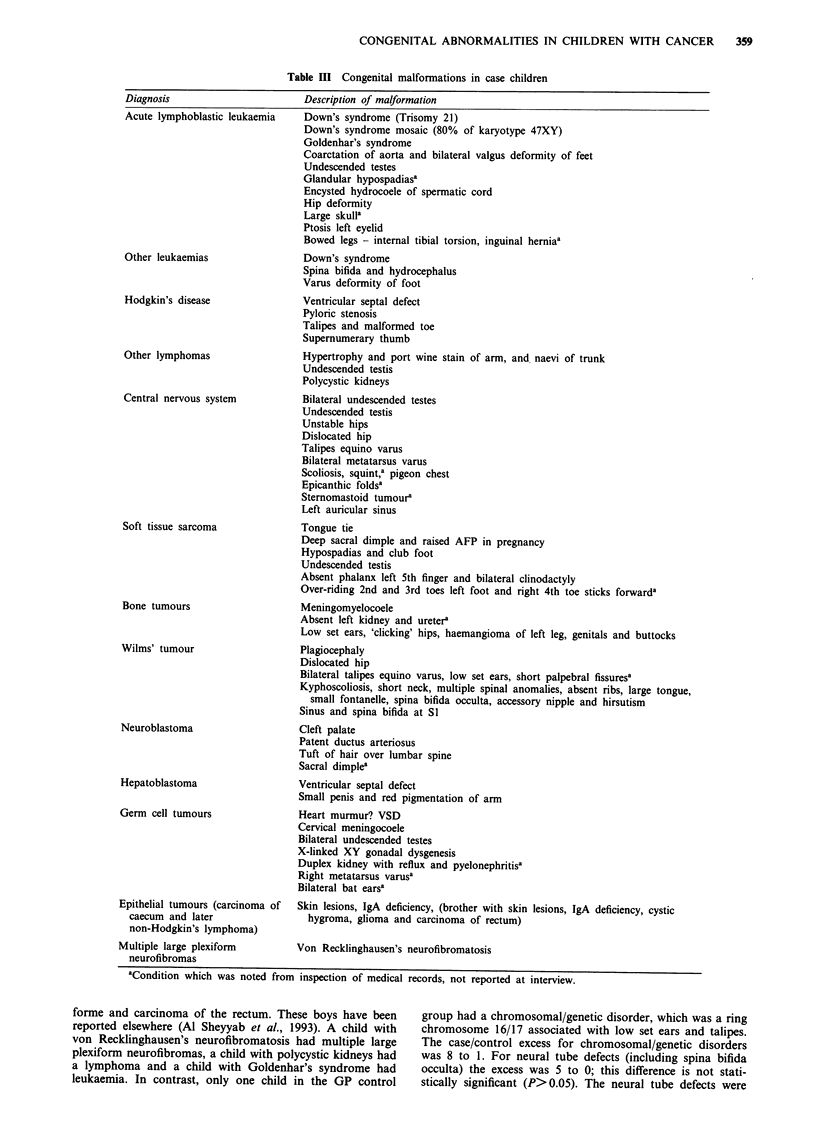

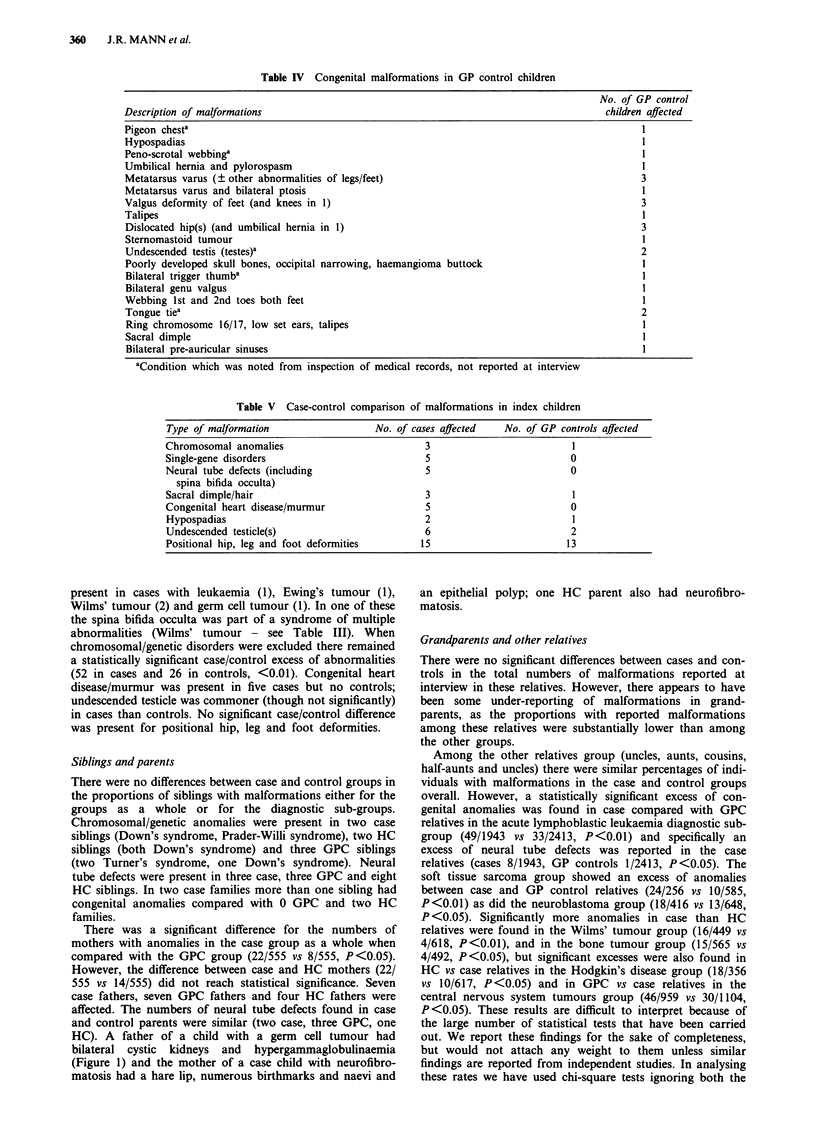

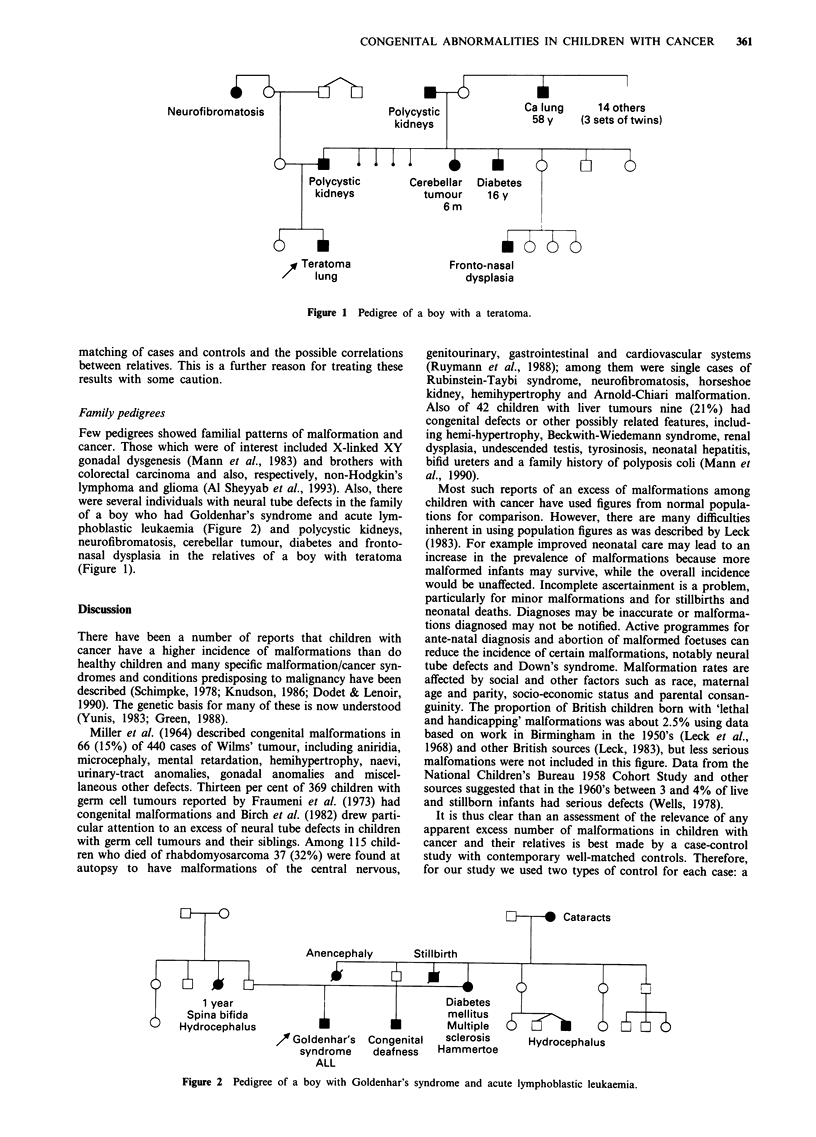

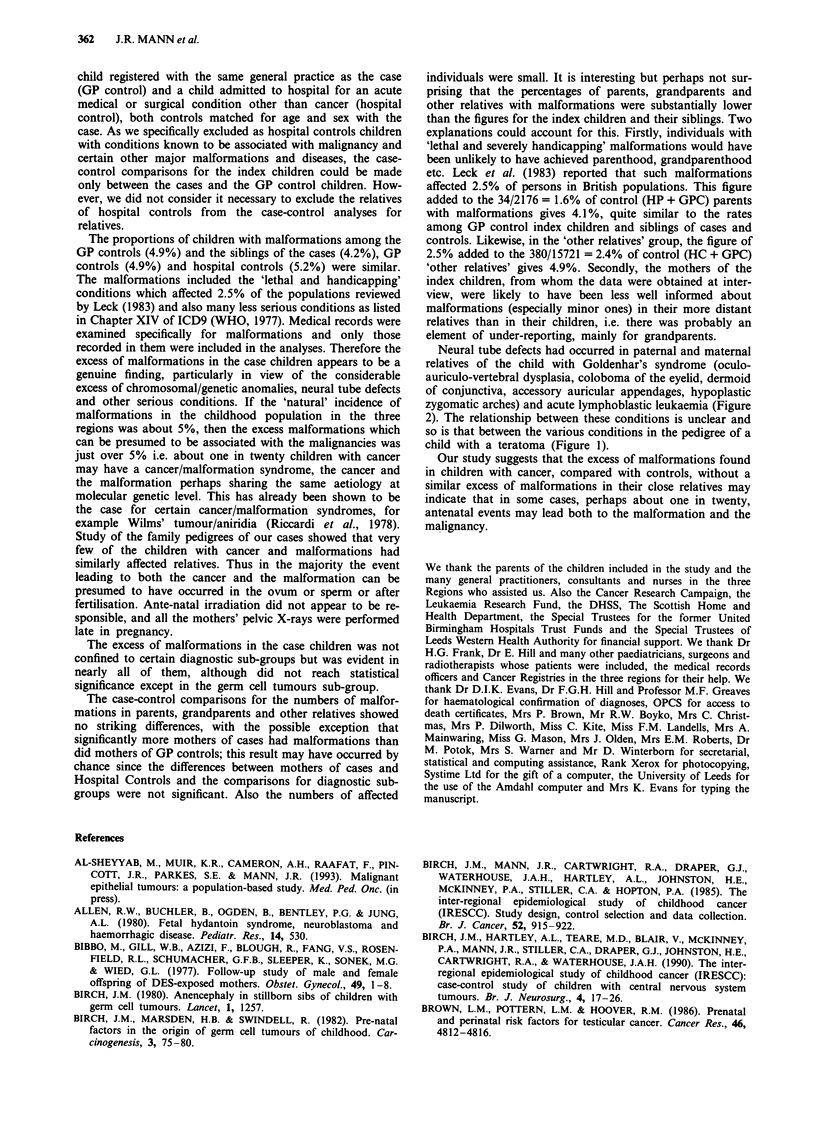

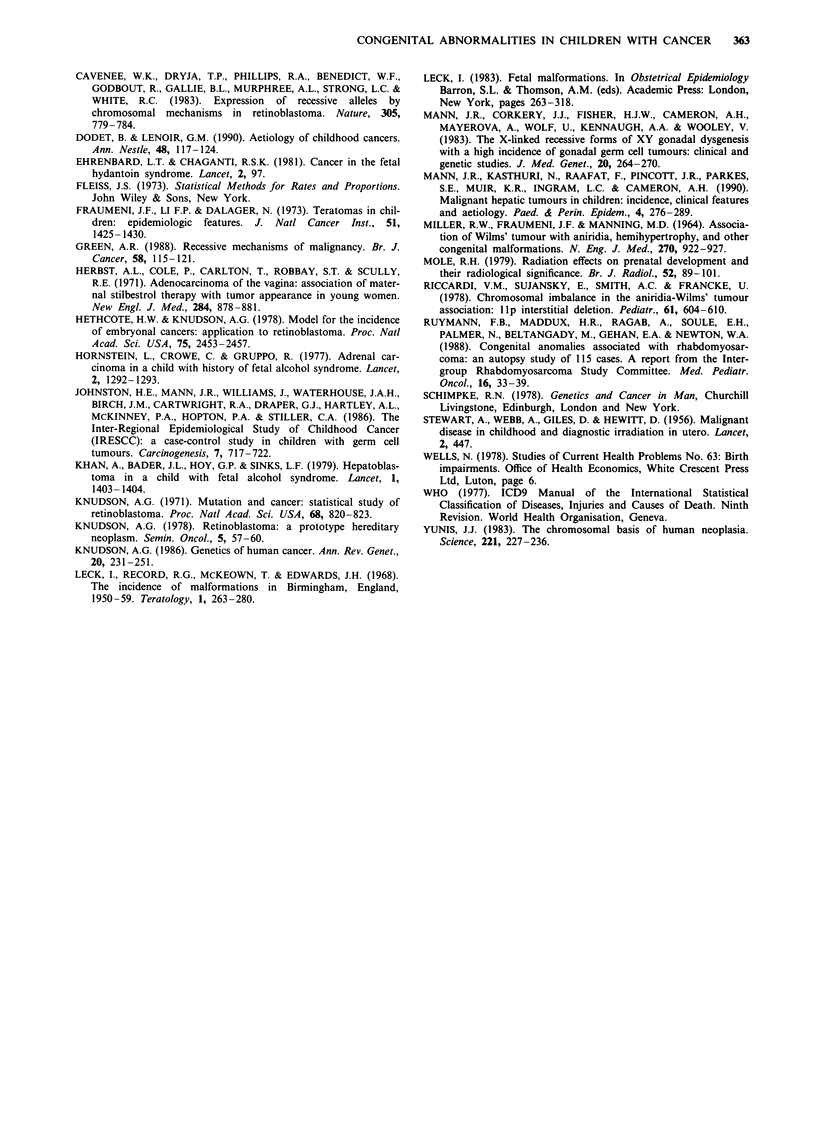


## References

[OCR_00822] Bibbo M., Gill W. B., Azizi F., Blough R., Fang V. S., Rosenfield R. L., Schumacher G. F., Sleeper K., Sonek M. G., Wied G. L. (1977). Follow-up study of male and female offspring of DES-exposed mothers.. Obstet Gynecol.

[OCR_00826] Birch J. M. (1980). Anencephaly in stillborn sibs of children with germ cell tumours.. Lancet.

[OCR_00843] Birch J. M., Hartley A. L., Teare M. D., Blair V., McKinney P. A., Mann J. R., Stiller C. A., Draper G. J., Johnston H. E., Cartwright R. A. (1990). The inter-regional epidemiological study of childhood cancer (IRESCC): case-control study of children with central nervous system tumours.. Br J Neurosurg.

[OCR_00835] Birch J. M., Mann J. R., Cartwright R. A., Draper G. J., Waterhouse J. A., Hartley A. L., Johnston H. E., McKinney P. A., Stiller C. A., Hopton P. A. (1985). The Inter-Regional Epidemiological Study of Childhood Cancer (IRESCO). Study design, control selection and data collection.. Br J Cancer.

[OCR_00830] Birch J. M., Marsden H. B., Swindell R. (1982). Pre-natal factors in the origin of germ cell tumours of childhood.. Carcinogenesis.

[OCR_00851] Brown L. M., Pottern L. M., Hoover R. N. (1986). Prenatal and perinatal risk factors for testicular cancer.. Cancer Res.

[OCR_00858] Cavenee W. K., Dryja T. P., Phillips R. A., Benedict W. F., Godbout R., Gallie B. L., Murphree A. L., Strong L. C., White R. L. Expression of recessive alleles by chromosomal mechanisms in retinoblastoma.. Nature.

[OCR_00869] Ehrenbard L. T., Chaganti R. S. (1981). Cancer in the fetal hydantoin syndrome.. Lancet.

[OCR_00877] Fraumeni J. F., Li F. P., Dalager N. (1973). Teratomas in children: epidemiologic features.. J Natl Cancer Inst.

[OCR_00976] GILES D., HEWITT D., STEWART A., WEBB J. (1956). Malignant disease in childhood and diagnostic irradiation in utero.. Lancet.

[OCR_00882] Green A. R. (1988). Recessive mechanisms of malignancy.. Br J Cancer.

[OCR_00886] Herbst A. L., Ulfelder H., Poskanzer D. C. (1971). Adenocarcinoma of the vagina. Association of maternal stilbestrol therapy with tumor appearance in young women.. N Engl J Med.

[OCR_00892] Hethcote H. W., Knudson A. G. (1978). Model for the incidence of embryonal cancers: application to retinoblastoma.. Proc Natl Acad Sci U S A.

[OCR_00897] Hornstein L., Crowe C., Gruppo R. (1977). Adrenal carcinoma in child with history of fetal alcohol syndrome.. Lancet.

[OCR_00902] Johnston H. E., Mann J. R., Williams J., Waterhouse J. A., Birch J. M., Cartwright R. A., Draper G. J., Hartley A. L., McKinney P. A., Hopton P. A. (1986). The Inter-Regional, Epidemiological Study of Childhood Cancer (IRESCC): case-control study in children with germ cell tumours.. Carcinogenesis.

[OCR_00910] Khan A., Bader J. L., Hoy G. R., Sinks L. F. (1979). Hepatoblastoma in child with fetal alcohol syndrome.. Lancet.

[OCR_00923] Knudson A. G. (1986). Genetics of human cancer.. Annu Rev Genet.

[OCR_00915] Knudson A. G. (1971). Mutation and cancer: statistical study of retinoblastoma.. Proc Natl Acad Sci U S A.

[OCR_00919] Knudson A. G. (1978). Retinoblastoma: a prototypic hereditary neoplasm.. Semin Oncol.

[OCR_00927] Leck I., Record R. G., McKeown T., Edwards J. H. (1968). The incidence of malformations in Birmingham, England, 1950-1959.. Teratology.

[OCR_00950] MILLER R. W., FRAUMENI J. F., MANNING M. D. (1964). ASSOCIATION OF WILMS'S TUMOR WITH ANIRIDIA, HEMIHYPERTROPHY AND OTHER CONGENITAL MALFORMATIONS.. N Engl J Med.

[OCR_00937] Mann J. R., Corkery J. J., Fisher H. J., Cameron A. H., Mayerová A., Wolf U., Kennaugh A. A., Woolley V. (1983). The X linked recessive form of XY gonadal dysgenesis with a high incidence of gonadal germ cell tumours: clinical and genetic studies.. J Med Genet.

[OCR_00944] Mann J. R., Kasthuri N., Raafat F., Pincott J. R., Parkes S. E., Muir K. R., Ingram L. C., Cameron A. H. (1990). Malignant hepatic tumours in children: incidence, clinical features and aetiology.. Paediatr Perinat Epidemiol.

[OCR_00955] Mole R. H. (1979). Radiation effects on pre-natal development and their radiological significance.. Br J Radiol.

[OCR_00959] Riccardi V. M., Sujansky E., Smith A. C., Francke U. (1978). Chromosomal imbalance in the Aniridia-Wilms' tumor association: 11p interstitial deletion.. Pediatrics.

[OCR_00964] Ruymann F. B., Maddux H. R., Ragab A., Soule E. H., Palmer N., Beltangady M., Gehan E. A., Newton W. A. (1988). Congenital anomalies associated with rhabdomyosarcoma: an autopsy study of 115 cases. A report from the Intergroup Rhabdomyosarcoma Study Committee (representing the Children's Cancer Study Group, the Pediatric Oncology Group, the United Kingdom Children's Cancer Study Group, and the Pediatric Intergroup Statistical Center).. Med Pediatr Oncol.

[OCR_00991] Yunis J. J. (1983). The chromosomal basis of human neoplasia.. Science.

